# Rare taxa modulate the emergence of dominants in microbial communities

**DOI:** 10.1128/mbio.02598-25

**Published:** 2025-11-25

**Authors:** Jianing Wang, Jianshui Yu, Zhuo Pan, Zheng Zhang, Yue-zhong Li

**Affiliations:** 1 State Key Laboratory of Microbial Technology, Institute of Microbial Technology, Shandong University 12589, Qingdao, China; 2 Shenzhen Research Institute, Shandong University, Shenzhen, China; Corporación CorpoGen, Bogotá D.C., Colombia

**Keywords:** microbial community assembly, dominant taxa, rare taxa, microcosms, consumer–resource model

## Abstract

Microbial communities typically comprise a few dominant taxa alongside numerous rare members. While dominant formation is generally attributed to species-level traits that confer adaptation to specific environments, it remains unclear how the rare biosphere influences which taxa ultimately become dominant. We constructed thousands of sub-communities from a soil microbiome using appropriate dilution to preserve the dominants but allow stochastic variation in rare assemblages. Following cultivation under an identical growth condition, dominant taxa varied substantially across replicates, which were determined to be driven primarily by species generalist competitive capacities rather than their initial relative abundances. Notably, the identity of the most abundant taxon in each sub-community was modulated by interactions with rare community members. Co-occurrence and metabolic resource overlap analyses revealed predominantly negative interactions among dominant taxa, while rare taxa exhibited variable and asymmetric interaction patterns. A refined consumer–resource model supported these observations, showing that species are first “nominated” as dominant candidates based on generalist competitive capacities, and then “voted” into the most abundant taxon in a community through collective feedback from rare taxa. The findings imply that dominant emergence in microbial communities is not solely determined by intrinsic species traits but is also critically shaped by interactions with rare taxa. The proposed “nomination–voting” model highlights a collective role of the rare biosphere in shaping community structure and offers a new framework for understanding microbial assembly.

**IMPORTANCE:**

Microbial ecosystems are almost always dominated by only a few species, but their diversity resides in the rare biosphere. These rare members are usually considered passive passengers with little influence, yet our work reveals that they can collectively determine which species to become the most abundant taxon. We describe this process as a “nomination–voting” system: competitive traits nominate potential dominants, while rare taxa vote for the ultimate winner through their complex interactions. Recognizing this hidden but decisive role of rare microbes provides a new perspective on community assembly and underscores how subtle ecological interactions shape community outcomes. This assembly framework offers new opportunities for the prediction, manipulation, and stabilization of agriculture, health, and environmental microbiomes.

## INTRODUCTION

Different habitats harbor different microbial communities, which almost always consist of numerous rare taxa together with a few dominants (1, 2). While dominant members are often responsible for key ecological functions and tend to recur across similar environments as core taxa, rare taxa contribute the majority of community diversity and exhibit highly variable compositions (1, 3–7). Microbial communities cultivated under identical conditions tend to converge in taxonomic composition (8–12), suggesting that dominant formation is primarily driven by species-level traits that confer adaptation to the given environment. However, it is unclear whether and how the rare biosphere affects the formation of dominant taxa.

The inherent complexity of natural microbial communities limits our understanding of community assembly processes, including how community structure forms and diversity is maintained. To address this, researchers have adopted two general strategies: the top-down approach decreases species diversity and interactions by artificially controlling culture conditions (8–12), while bottom-up approaches assemble synthetic microbial communities from defined isolates (13–15). However, conflicting outcomes are sometimes observed—for example, the inconsistency between species-pair coexisting rules and the coexistence of multispecies in a complex community (13, 16, 17). These inconsistencies highlight that over-simplification can eliminate the subtle but potentially influential roles of rare taxa. To probe the mechanisms underlying dominant formation, an approach is to retain core ecological structure while allowing controlled variation in rare community members.

**Fig 1:**
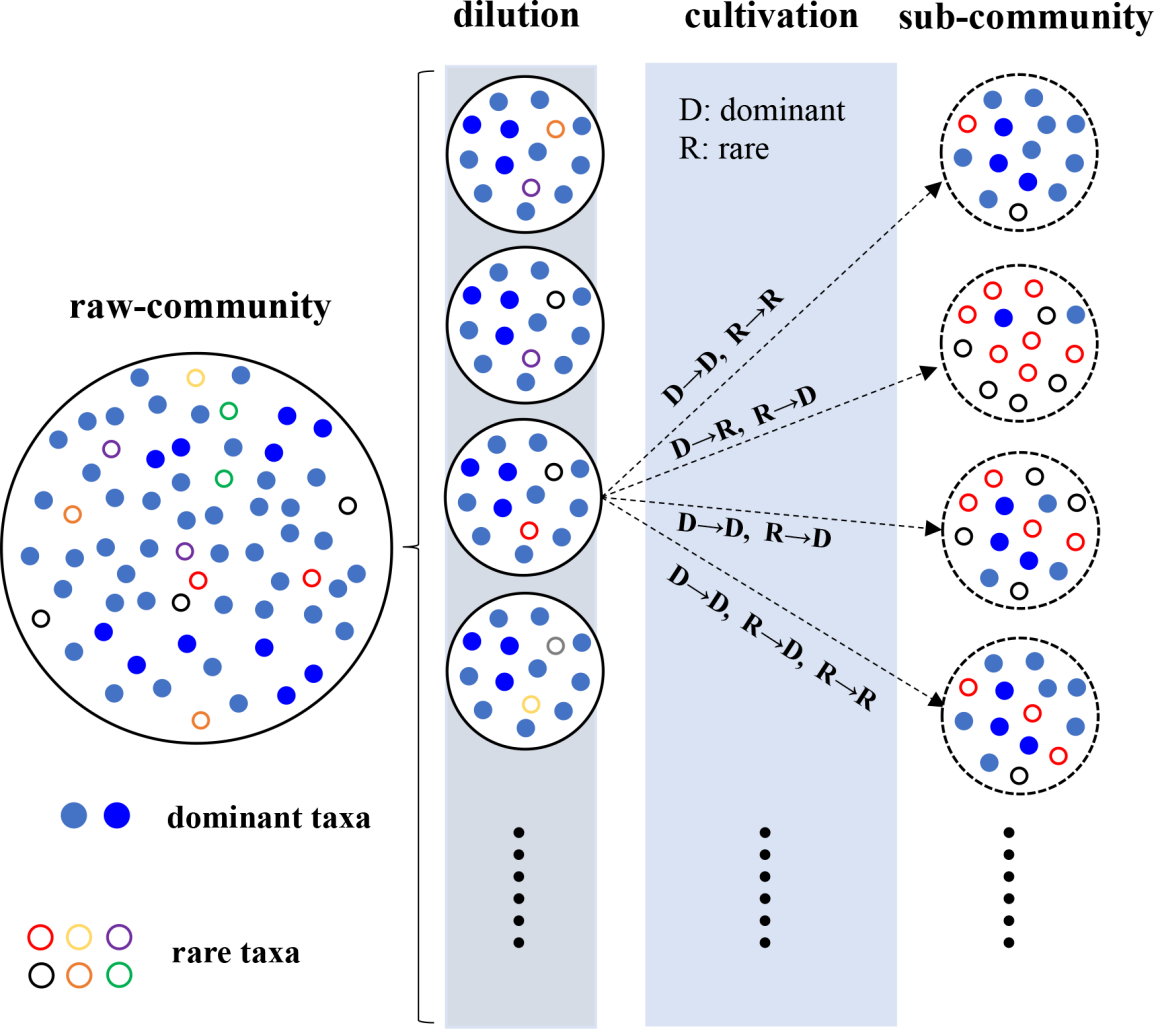
A sketched inference for the dilution at the complexity threshold and potential compositions of sub-communities after cultivation.

In a sense, dilution is the simplest and most viable method for simplifying microbial communities (9, 11, 18, 19). Dilution is unspeciﬁc, and the occurrence of microorganisms in a diluted subset theoretically depends on their initial abundance; low dilution allows the presence of most raw taxa, while high dilution results in the loss of most taxa. By appropriately diluting a natural soil microbiome, we generated sub-communities in which dominant taxa are retained, whereas rare taxa are randomly lost or preserved. This design allows us to construct replicate communities with similar pools of dominant candidates but variable rare assemblages, providing an ideal system to test whether and how the rare biosphere affects dominant formation under uniform environmental conditions (Fig. 1).

In this study, we cultivated over 900 sub-communities derived from a single soil microbiome and observed substantial variation in dominant taxa across replicates. We characterized the ecological traits of dominants and found them to be generalist competitors with fast growth rates and high metabolic flexibility. To mechanistically evaluate the role of rare taxa, we further developed an improved consumer-resource model that simulated community assembly with fixed dominant candidates and randomized rare backgrounds. Both experimental and model results support a “nomination and voting” framework, in which dominant candidates are nominated by their intrinsic traits, while the final dominant—the most abundant taxon—is determined in a community through collective interactions with rare community members.

## RESULTS

### Variation of sub-communities after dilution-cultivation

The soil microbial community sample for the dilution and cultivation study was collected from the rhizosphere of wheat seedlings (*Triticum aestivum*) grown on cultivated land in the Yellow River Delta wetlands. Estimated from about 50,000 reads of the 16S rRNA gene amplicons (in triplicate), the number of observed operational taxonomic units (OTUs) in the raw community was 674. To justify the sequencing depth, we generated rarefaction curves for the raw community, which approached saturation at 50,000 reads (Fig. S1a), indicating that this depth is sufficient to capture its diversity. In this study, we set the relative abundance of 1.0% as the threshold to designate the dominant and rare OTUs, and the 16 dominant OTUs accounted for 68.59% of the total abundance in the raw community (Fig. S2; details refer to Table S1).

After pretests, three dilutions, that is, 1.0 × 10^−4^, 0.5 × 10^−4^, and 1.0 × 10^−5^, were cultivated in 10% tryptic soy broth (TSB) medium on the 96-well plate, resulting in a total of 1,056 sub-communities. The 16S rRNA gene amplicons of each sub-community were sequenced, and those with fewer than 5,000 reads were removed (Fig. S1b, indicating that 5,000 reads is a threshold that balances diversity retention and sample size), resulting in 908 sub-communities (Fig. S3) for further analysis. The median values of the observed OTUs decreased to 35 in sub-communities and 59, 33, and 24 for the 1.0 × 10^−4^, 0.5 × 10^−4^, and 1.0 × 10^−5^ dilutions, respectively (Fig. S4). We found that the compositions of sub-communities were greatly diverse after cultivation (Table S2 provides the sequencing details of the 908 sub-communities), in accordance with the varied alpha diversity indices (Table S3).

**Fig 2:**
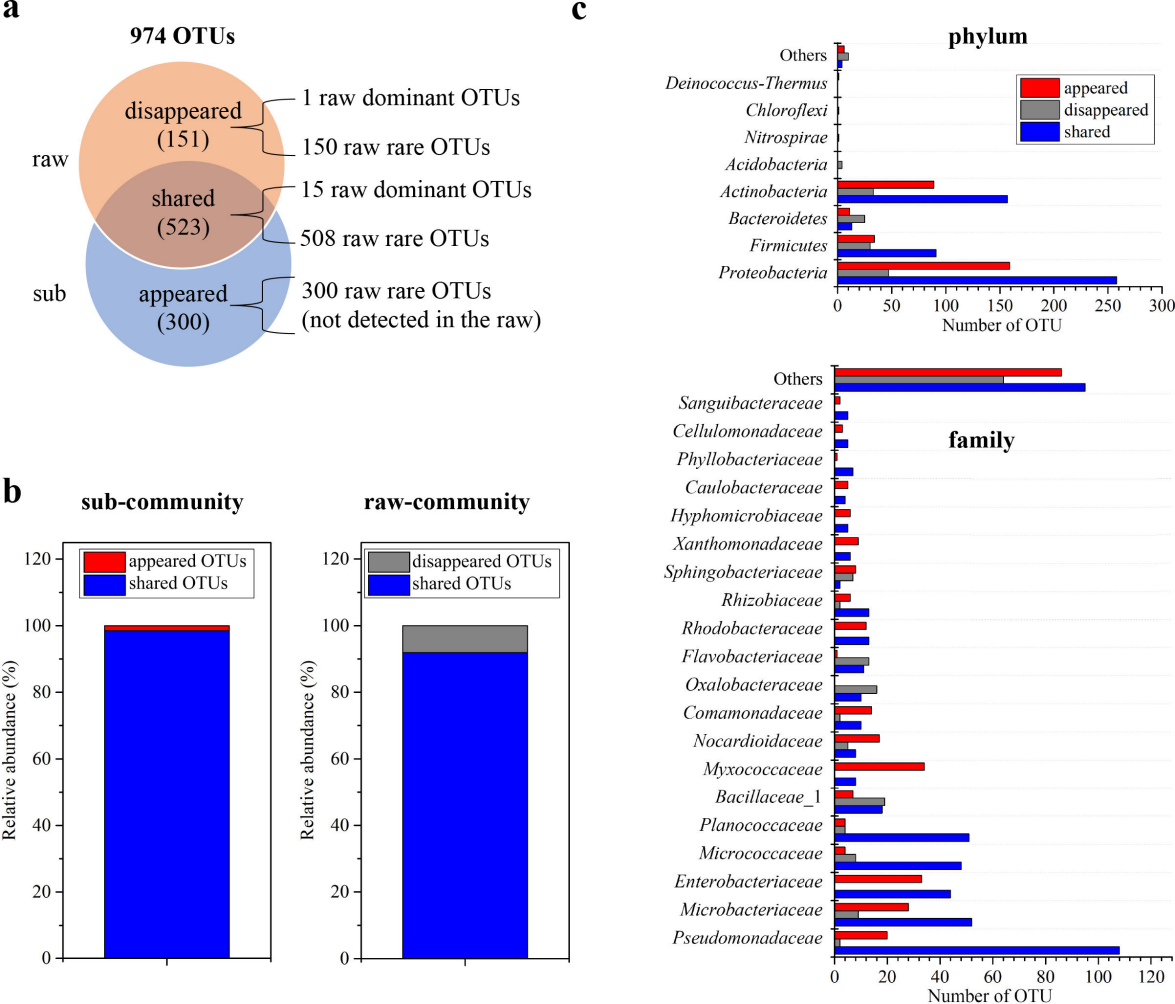
Comparisons of microbial communities before and after dilution cultivation. The number (**a**), relative abundance (**b**), and taxonomic compositions (**c**) of shared, appeared, and disappeared OTUs in raw community and sub-community.

Compared to the raw community, many new OTUs “appeared” in sub-communities, in addition to the “shared” (present in both raw community and sub-community) and “disappeared” OTUs (present in raw community but not sub-communities; Fig. 2a; Table S4), suggesting that many extremely rare OTUs were undetected by the amplicon sequencing but “retrieved” by dilution and large-scale cultivation. Although the shared, disappeared, and appeared parts each contained many observed OTUs, the relative abundance of the shared OTUs, being 91.88% in the raw community, occupied 98.49% in the total sub-communities (Fig. 2b), suggesting an overall consistency between the raw community and sub-communities after the dilution and cultivation. We noticed that the shared, disappeared, and appeared OTUs were taxonomically distinct, and the members within a high taxonomic unit might appear in different parts (Fig. 2c demonstrates tracks of different taxonomic units at the phylum and the family levels in the three parts; details refer to Fig. S5; Table S5). For instance, *Proteobacteria* is the most abundant phylum, and the number of OTUs of this phylum in the disappeared, shared, and appeared parts were 47, 258, and 159, respectively; the number of OTUs of the most abundant family *Pseudomonadaceae* was 2, 108, and 20 in the disappeared, shared, and appeared parts, respectively.

### Turnover of OTU status in sub-communities

**Fig 3:**
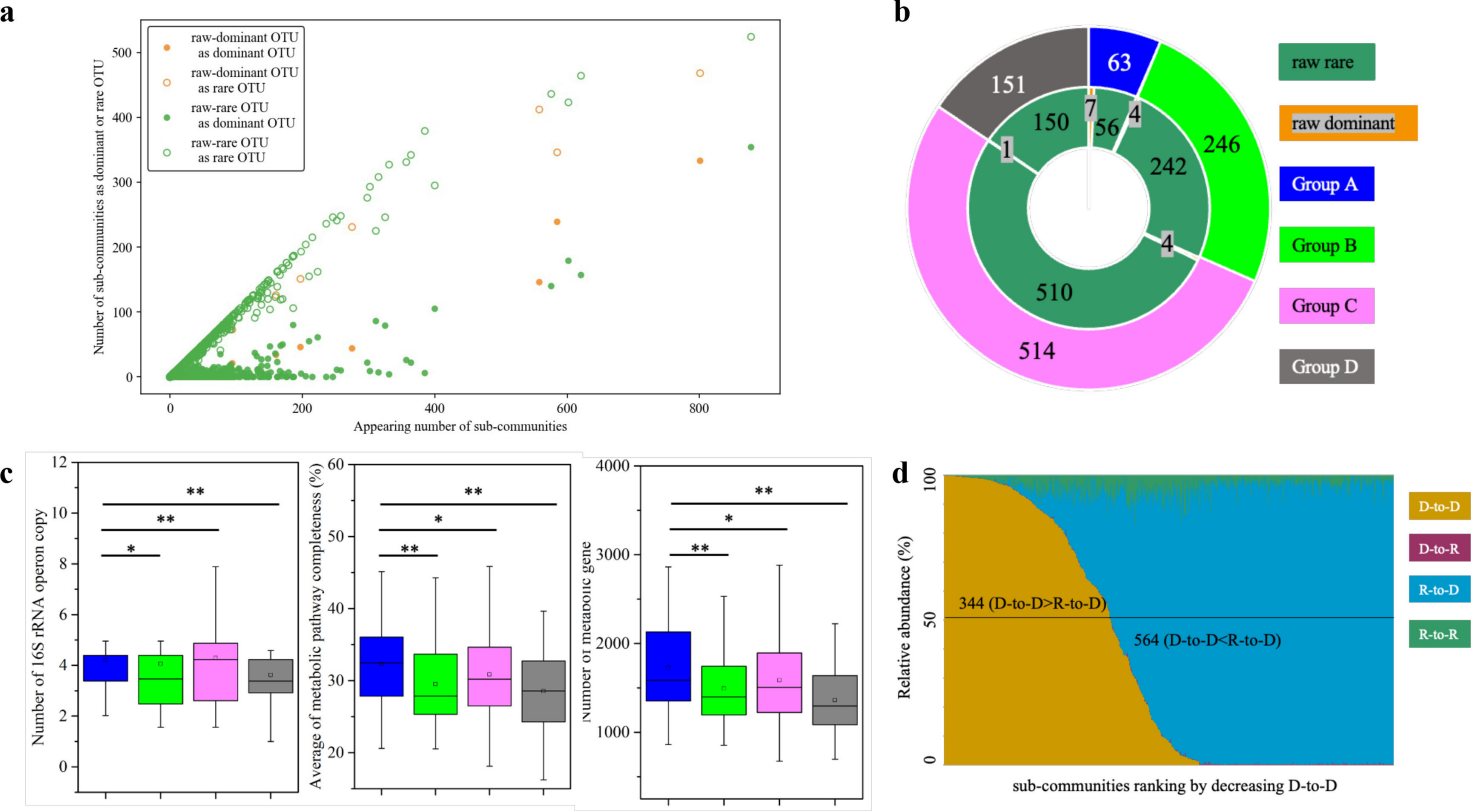
Occurrence and contribution factors for the OTUs in sub-communities. (**a**) Relationships between the appearing number and the frequency to become dominant or rare in sub-communities. (**b**) The fate of OTUs from raw communities to sub-communities. (**c**) Comparison of 16S rRNA operon copy number (calculated at the family level), metabolic gene number, and the average metabolic pathway completeness among the OTUs in different groups. **: *P* < 0.01, *: *P* < 0.05, Wilcoxon test. (**d**) Changes in the relative abundance of D-to-D, R-to-D, D-to-R, and R-to-R OTUs, along with the relative abundance of D-to-D OTUs. D-to-D: the OTUs from dominant to dominant; R-to-D: rare to dominant, D-to-R: dominant to rare, R-to-R: rare to rare.

Based on the threshold of 1.0% relative abundance, lots of raw-dominant or raw-rare OTUs became dominant with different frequencies in sub-communities (Table S6). For example, OTU_1 was dominant, and OTU_2 was rare in the raw community, having the relative abundances of 26.24% and 0.02%, respectively; however, they appeared in 801 and 878 sub-communities: 333 and 354 times as the dominant, and 468 and 524 times as the rare, respectively. We noticed that there was a positive relationship between the appearing number and the frequency to become dominant or rare in sub-communities for either the raw-dominant or the raw-rare OTUs (Fig. 3a; results with alternative thresholds are shown in Fig. S6), which suggested that becoming dominant or rare of an OTU, as well as its appearing frequency, is not driven solely by its initial relative abundance in the raw community.

We classified the sub-community OTUs into four categories: appearing as dominant in 10 or more sub-communities (group A), in 1–9 sub-communities (group B), never being dominant in sub-communities (group C), and disappearing in sub-communities (group D). These categories included both the raw-dominant and the raw-rare OTUs (Fig. 3b, the newly appeared OTUs were taken as the raw-rare). For example, among the 63 group-A OTUs, 7 were from the raw dominant, and 56 were from the raw rare. Notably, among the 300 newly appeared OTUs, 4 were in group A, 45 were in group B, and 251 were not dominant in any sub-community (group C).

To evaluate the species eco-competitive capacities of the OTUs in the above four categories, we analyzed their 16S rRNA operon copies (20, 21), metabolic gene number (22), and metabolic pathway completeness (23). Interestingly, all these generalist eco-competitive characteristics were significantly higher in the group-A OTUs than those of the other categories (Fig. 3c; detailed data were listed in Table S6). Thus, the intrinsic competition capacities determine in large the tendency of an OTU to become dominant or rare in communities, that is, compared to OTUs of other categories, the group-A OTUs generally exhibit greater capacities for fast growth, more complete metabolic pathways, and higher metabolic flexibility.

The dominant-to-dominant (raw-dominant being dominant in sub-communities; D-to-D) and rare-to-dominant (R-to-D) OTUs each had a high relative abundance in sub-communities, occupying 35.64% and 60.75% of the total, respectively, while the abundance contributions of the dominant-to-rare (D-to-R) and rare-to-rare (R-to-R) OTUs together accounted for only 3.61% (Fig. 3d; Table S7), indicating that dominant OTUs, including D-to-D and R-to-D, are obviously the major contributors to the relative abundance in sub-communities. In contrast, the R-to-R OTUs constitute the majority of the biodiversity across sub-communities (Fig. S7), and the Spearman’s *r* values for the correlation of observed OTUs with the number of R-to-R OTUs were 0.99, followed by R-to-D (0.69), D-to-R (0.38), and D-to-D (0.16; Fig. S8).

### Patterns and ecological drivers of OTU coexistence

**Fig 4:**
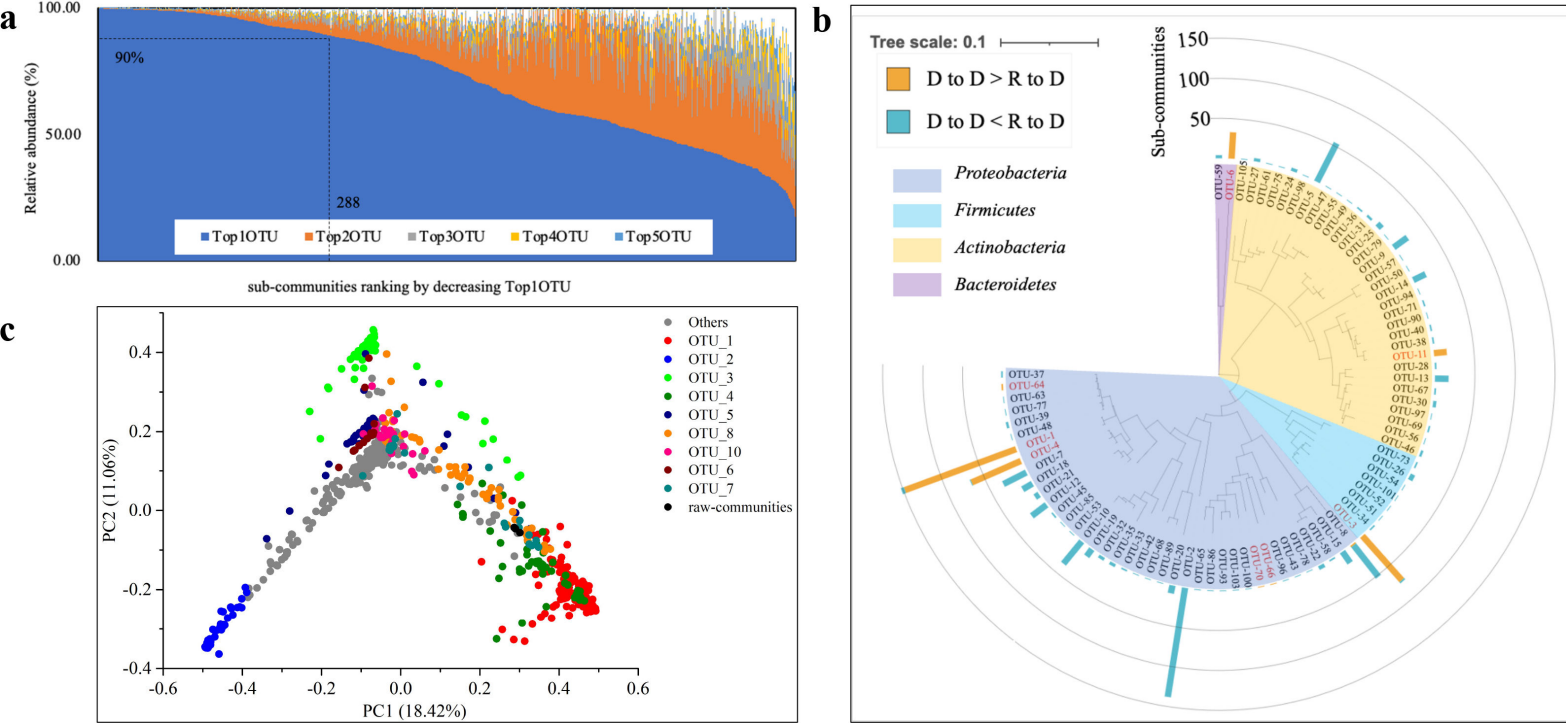
Coexistence and interactions of OTUs in sub-communities. (**a**) The POC of OTU pairs. (**b**) Interaction dynamics of OTU pairs. (**c**) The MRO of OTU pairs in sub-communities. OTU pairs: DD_coe_ (dominant-dominant), DR_coe_ (dominant-rare), RD_coe_ (rare-dominant), and RR_coe_ (rare-rare). **: *P* < 0.01, *: *P* < 0.05, Wilcoxon test.

To further investigate how rare taxa influence dominant formation, we examined the co-occurrence patterns among OTUs across sub-communities. We wondered whether OTUs tend to co-occur or exclude one another, and whether such patterns reflect ecological constraints such as metabolic resource overlap (MRO). We calculated the pairwise possibility of coexistence (POC) between OTUs, a metric reflecting whether the presence of one OTU increases (POC > 1), decreases (0 < POC < 1), or has no effect (POC = 1) on the occurrence of another across sub-communities. OTU pairs with POC = 0 or +∞ were considered to show complete exclusion or complete coexistence, respectively (Fig. S9). This analysis assumes that all OTUs above 0.01% relative abundance in the raw community were statistically retained in all sub-communities after dilution, and their observed co-occurrence patterns reflect emergent ecological interactions under uniform environmental conditions. To reduce stochastic noise, we focused on 169 OTUs that appeared in more than 10 sub-communities, resulting in 28,392 pairwise comparisons. Among these OTU pairs, we observed a pronounced skew toward exclusionary co-occurrence patterns: 11.65% were completely exclusive (POC = 0), and 80.86% showed exclusion preference (0 < POC < 1). In contrast, only 6.83% of pairs showed coexistence preference (POC >1), 0.34% were completely coexisting (POC = +∞), and 0.32% were neutral (POC = 1; Fig. 4a; Table S8). Although POC may be affected by dilution-induced stochasticity, our large number of replicates and strict filtering mitigate this uncertainty. As a descriptive, occurrence-based metric, POC is therefore interpreted as consistent with, rather than definitive proof of, a competition-dominated assembly.

Notably, the coexistence of two OTUs was frequently asymmetrical. For example, OTU_1 occurred in 801 of the 908 sub-communities, OTU_13 was found in 151 sub-communities, and both were present in 125 sub-communities. Consequently, the POC value was 0.18 for OTU_13 to OTU_1 but was 4.81 for OTU_1 to OTU_13. The POC value ≥ 1 suggests that an OTU has a positive effect on the coexistence of the other, while a value of 1 > POC value ≥0 suggests a negative effect. For the OTU_1 and OTU_13 pair, their interaction type is “positive + negative.” There are four possible interaction types for two OTUs: “positive + positive,” “positive + negative,” “negative + positive” and “negative + negative,” accounting for 0.99%, 0.68%, 12.33%, and 86.00% of all the analyzed OTU pairs, respectively (Fig. 4b). Obviously, the interactions between two OTUs were predominantly negative, which is consistent with the previous suggestion that cooperation among bacteria is rare (16), that is, OTUs in a microbial community mostly tend to exclude one another; however, it may also be accentuated under the nutrient-rich TSB medium, which preferentially supports fast-growing, competitive taxa.

To explore the ecological basis of exclusion, we calculated the MRO between OTU pairs, which quantifies the similarity in their nutritional requirements (24). We mapped the 16S rRNA gene sequences of all the observed 823 OTUs to their closest reference genomes in the National Center for Biotechnology Information (NCBI) RefSeq database (25) with a 97% identity and obtained 555 matching genomes. Compared to that of random OTU pairs, the MRO value had no significant differences for the coexisting dominant and rare OTUs or rare and rare OTUs, but exhibited significantly higher values for the coexisting dominant and dominant OTUs (*P* < 0.01, Wilcoxon test; Fig. 4c; Table S9). This suggests that exclusion among dominant taxa is driven by deterministic niche overlap, while coexistence involving rare taxa is more likely shaped by stochastic processes. These results support the view that dominant OTUs, characterized by strong competitive traits and overlapping metabolic niches, are generally incompatible with one another. In contrast, rare OTUs may either coexist randomly or subtly modulate community outcomes through weak or asymmetric interactions.

### Emergence and characteristics of Top1OTUs across sub-communities

**Fig 5:**
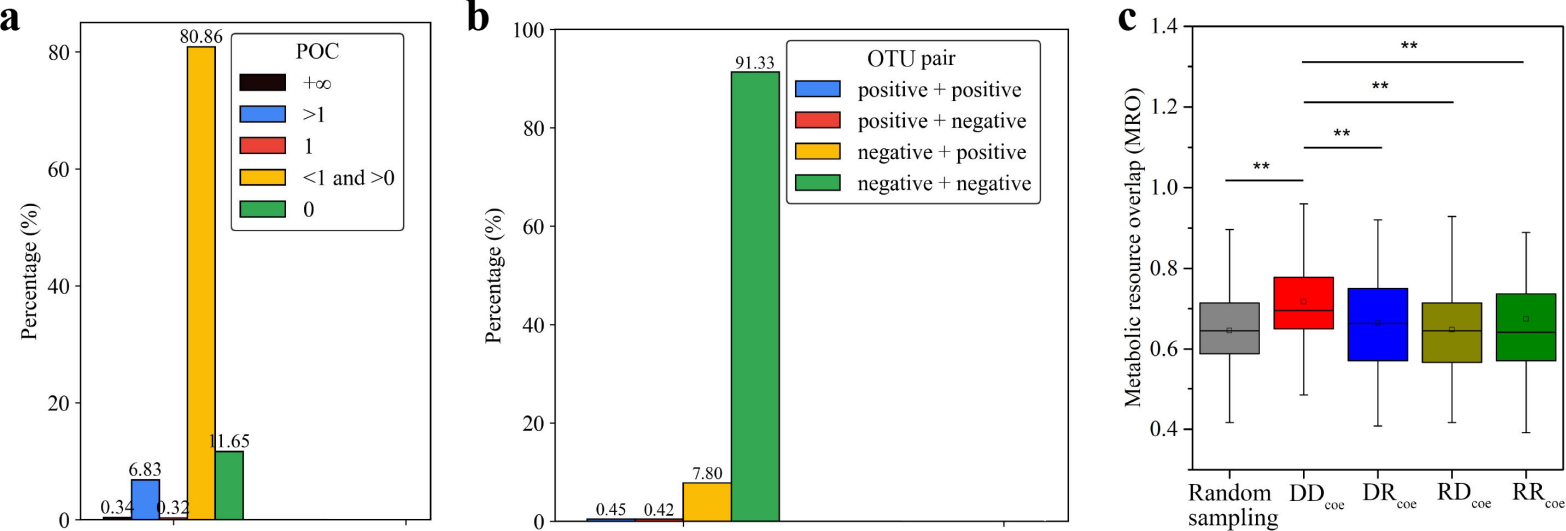
Emergence and characteristics of Top1OTUs across sub-communities. (**a**) Relative abundance of Top1 to Top5 OTUs in sub-communities. (**b**) Phylogenetic analysis of the 81 Top1OTUs appeared in sub-communities. The red label indicates the raw-dominant taxa, and the black label indicates the raw-rare taxa. (**c**) Principal coordinate analysis based on Bray-Curtis metric results (OTU level) of the sub-communities with highlights of the major Top1OTUs. **: *P* < 0.01.

Large-scale cultivation of diluted soil microbiota substantially simplified community structures but also led to the emergence of strikingly uneven dominance patterns. Across the 908 sub-communities, over 80% had only 1–6 dominant OTUs (Fig. S10), and many were dominated by a single OTU with exceptionally high abundance (Fig. S11). This steep dominance hierarchy was further reflected in the rapid decline in abundance from the top-ranked OTU (Top1OTU) to the second through fifth (Top2–Top5), where few Top2 OTUs exceeded 10% relative abundance, and none of the Top5 OTUs surpassed this threshold (Fig. 5a; Table S10). Such community structures enable tractable dissection of Top1OTU emergence by minimizing the confounding influence of other dominant taxa.

Despite uniform environmental and nutritional conditions, the identity of Top1OTUs varied considerably across replicates. In total, 81 distinct OTUs were identified as the Top1OTUs in at least one sub-community. These OTUs spanned four major bacterial phyla—*Proteobacteria*, *Firmicutes*, *Actinobacteria*, and *Bacteroidetes*—highlighting phylogenetic diversity and ecological divergence (Fig. 5b ; Table S11). These Top1OTUs exhibited a broad range of relative abundances—from 17.46% to 99.92%—indicating diverse levels of dominance strength (Table S10). While a few OTUs (e.g., OTU_1, OTU_2, and OTU_3) recurrently dominated large numbers of sub-communities (152, 138, and 76 times, respectively), a notable fraction—36 OTUs—attained Top1 status in only a single sub-community. The origins of these Top1OTUs in the raw community were highly skewed: only 8 Top1OTUs were raw-dominant, whereas 73 originated from raw-rare taxa, accounting for 349 and 559 dominant occurrences, respectively. We also found that the Top1OTUs were mostly from raw dominants in those sub-communities with higher relative abundance of D-to-D than R-to-D OTUs (termed D-to-D sub-communities; 342 of 344) but were mostly from raw rare in the R-to-D sub-communities (higher relative abundance of R-to-D than D-to-D; 557 of 564; Fig. 3d; details refer to Table S12).

Principal coordinate analysis revealed that sub-communities formed distinct clusters according to their Top1OTU identity (Fig. 5c; pairwise permutational multivariate analysis of variance (PERMANOVA) analysis results are listed in Table S13). Among sub-communities, the three most frequent Top1OTUs—OTU_1, OTU_2, and OTU_3—exhibited progressive shifts toward distinct vertices in ordination space as the relative abundance of the respective OTU increased (Fig. S12). Notably, two of the three most frequent Top1OTUs (OTU_2 and OTU_3) originated from rare backgrounds, suggesting that stochastic retention of rare taxa may influence which taxon ultimately becomes Top1OTU.

These results indicate that Top1OTU emergence is not solely determined by intrinsic traits such as growth or metabolic breadth but is also critically shaped by extrinsic factors—particularly the composition and interactions of rare taxa within the community. These context-dependent dynamics support a “nomination and voting” model, in which candidate dominants are nominated by their eco-competitive traits and ultimately “voted” through interaction-mediated feedback from rare members.

### Simulation-based validation of the nomination–voting model

To mechanistically validate the nomination–voting model proposed for dominant taxon emergence, we constructed a two-phase resource–consumer simulation framework. This model quantifies how species differing in eco-competitive capacities interact with their environments and with rare background taxa during community assembly. The two phases correspond to the “nomination” process—based on intrinsic traits—and the “voting” process—mediated by extrinsic community context.

In the first simulation, we examined whether species with higher eco-competitive capacities—namely faster growth rates and greater metabolic flexibility—were more likely to emerge as dominants during community assembly under resource-limited conditions. We initialized four functional groups of microbes: (i) high growth + high metabolic flexibility (HH), (ii) high growth + low flexibility (HL), (iii) low growth + high flexibility (LH), and (iv) low growth + low flexibility (LL), with 20 species per group (80 in total). Simulated communities were assembled under four resource conditions (1, 5, 10, or 20 resource types), with 100 replicates per condition.

**Fig 6:**
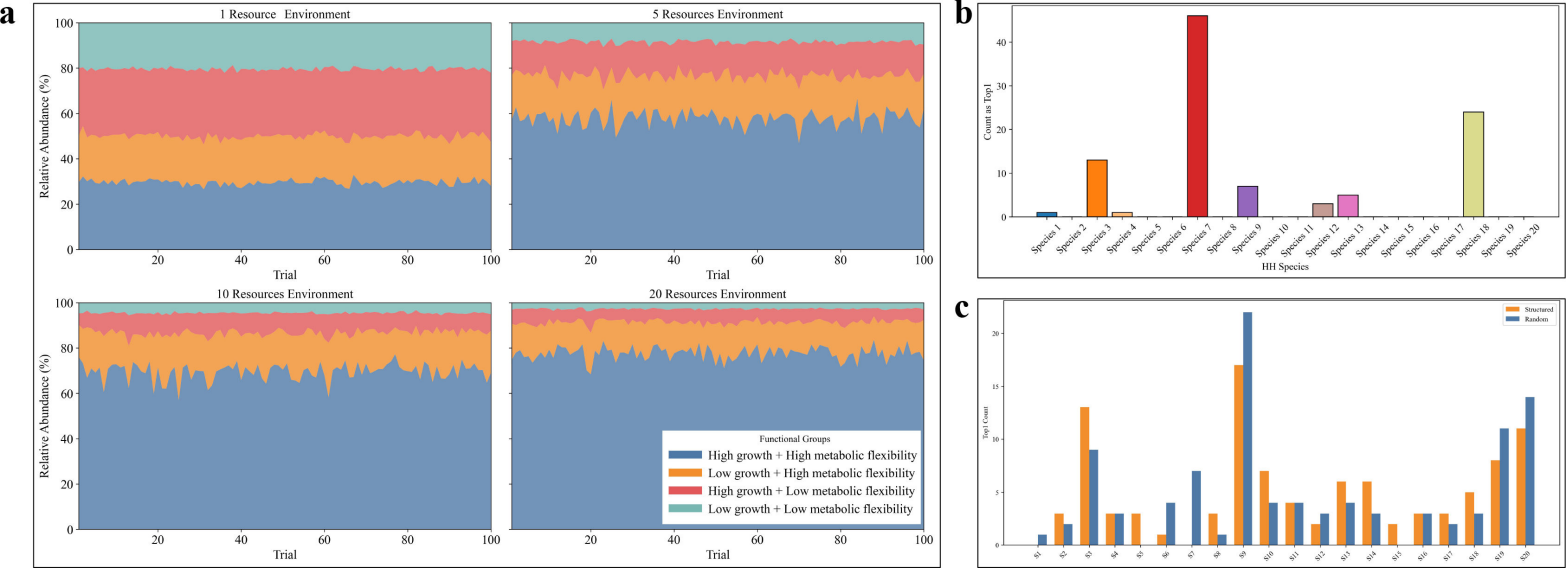
Simulation results of the resource–consumer model under the nomination–voting framework. (**a**) Relative abundances of four functional groups across 100 simulated communities under increasing resource richness (1, 5, 10, and 20 resources). Functional groups are defined by combinations of growth rate and metabolic flexibility. (**b**) Frequency distribution of how often each HH species became the most abundant taxon (Top1) across the 100 trials. (**c**) Structured rare taxa shift the distribution of Top1 outcomes across HH species compared with a random rare background.

The results consistently showed that HH species dominated the community, especially as resource complexity increased (Fig. 6a). In resource-rich environments, HH species accounted for the majority of total relative abundance, indicating that species with strong eco-competitive capacities are more likely to be “nominated” as candidate dominants. These findings validate the first half of the nomination–voting model: ecological competitiveness confers an advantage in community assembly and dominant potential.

We next conducted a second simulation to evaluate the influence of rare background taxa—that is, the “voting” process—on the emergence of the most abundant taxon. We fixed the species pool to include 20 HH “candidate” species whose total abundance accounted for 95% of the community. The remaining 5% was composed of 60 background species randomly drawn from the HL, LH, and LL groups, representing rare and ecologically distinct taxa. Simulations were conducted under a 10-resource environment with 100 replicates.

Although all HH species shared strong eco-competitive advantages, the identity of the most abundant taxon varied markedly across simulation replicates (Fig. S13). Different HH species emerged as dominant in different simulated communities, and the frequency with which each HH species achieved Top1 status showed substantial variability (Fig. 6b). These outcomes indicate that dominance was not only determined by intrinsic capacities but also by the specific composition of rare background taxa. Despite their low abundance, these rare members exerted “voting power” by modulating competitive dynamics through indirect interactions such as resource overlap or niche shielding. To further test this mechanism, we introduced a “structured rare taxa” scenario in which rare taxa were selectively biased to share resource-use overlap with certain dominant candidates. Compared with a random rare background, this structured configuration shifted the Top1 distribution across HH species, indicating that targeted overlap among rare taxa can modulate dominant formation (Fig. 6c).

The two simulations support our central hypothesis: the formation of dominants in microbial communities relies on a nomination–voting mechanism. Species with strong eco-competitive capacities are preferentially nominated, but the eventual emergence of a single dominant (Top1OTU) is contingent upon its interaction with co-occurring rare taxa. These rare members, by modulating resource access and interaction networks, effectively “vote” on which candidate taxon succeeds. This mechanism mirrors our experimental observations and offers a theoretical foundation for the context-dependent nature of microbial dominance.

## DISCUSSION

Understanding how dominant taxa emerge and persist during microbial community assembly remains a central question in microbial ecology, yet it is largely unresolved due to the high compositional diversity and complex ecological interactions within microbial communities. Natural microbial communities often harbor numerous functionally redundant species, which provide ecological buffering across diverse environmental conditions (26). While dominant members are typically assumed to drive key ecological functions, the mechanisms underlying their emergence are obscured by superimposed interactions among coexisting dominants. Previous studies have gained insights using bottom-up or top-down simplification strategies, such as synthetic communities constructed from defined isolates (13) or statistical models derived from closed systems (27). However, these approaches often oversimplify interaction networks and lack scalability for predicting high-order interactions or community outcomes in natural ecosystems (8). In particular, the influence of rare taxa on dominant formation has remained poorly understood.

To address this gap, we designed a dilution-based experimental framework to systematically examine how rare community members influence the emergence of dominant taxa. Specifically, we cultivated over 900 sub-communities derived from a single soil microbiome under an appropriate dilution regime. This approach preserved a relatively consistent pool of dominant candidates across sub-communities while introducing stochastic variation in the composition of rare taxa. This setup provided a unique opportunity to examine the influence of rare taxa on dominant outcomes under controlled conditions. Despite uniform environmental conditions and a shared pool of dominant candidates, we observed striking variation in the identity of the most abundant OTU (Top1OTU) among sub-communities. In total, 81 distinct OTUs emerged as Top1OTU in at least one replicate, with over 90% originating from rare taxa in the raw community. This high Top1 diversity suggests that rare taxa—though typically low in abundance—can substantially shape community trajectories and final dominant outcomes.

Mechanistically, we found that species more likely to become dominant consistently exhibited high eco-competitive capacities, including fast growth rates, broad metabolic flexibility, and more complete metabolic pathway profiles. These characteristics align with their role as dominant candidates adapted to the cultivation environment. However, the identity of the final Top1 was not solely determined by these traits. Co-occurrence analysis based on pairwise POC revealed that interactions among OTUs were predominantly negative, supporting the view that microbial interactions are largely competitive with limited cooperation (16, 17). It is important to note that our POC analysis assumes that all OTUs with relative abundance above 0.01% in the raw community are statistically retained across sub-communities after dilution. Because dilution is inherently stochastic, some absences may result from random loss rather than true ecological exclusion, which could lead to an overestimation of negative interactions. Nevertheless, several lines of evidence indicate that our main conclusion is robust. First, the large number of replicated sub-communities substantially reduces stochastic uncertainty and allows reliable estimation of co-occurrence frequencies. Second, the strong and consistent signal of predominantly negative POC values across independent analyses suggests a true ecological trend rather than a statistical artifact. Third, the congruent results from the MRO analysis—showing deterministic niche overlap among dominant taxa—further support the interpretation that competition, rather than random loss, primarily drives the observed exclusion patterns. This competition-dominated regime was likely intensified by the use of TSB, a nutrient-rich medium that promotes rapid growth and reduces niche differentiation, thereby favoring direct competition over cooperative interactions. While this setting provides a stringent testbed for studying competitive filtering, it may underestimate the complexity of interactions—including facilitation—that can emerge under nutrient-limited or spatially structured environments (16, 28, 29).

To further validate our conceptual framework, we developed a refined two-step resource–consumer model (30, 31) designed to separately assess the roles of intrinsic traits and community context. In the first step, species with high eco-competitive capacities were consistently enriched across a range of resource conditions, validating their nomination as dominant candidates. In the second step, we fixed the pool of candidates but varied the composition of rare background taxa. Despite identical trait profiles among candidates, their success in becoming Top1 varied significantly across replicates. These findings support a “nomination–voting” model, in which dominant candidates are nominated by trait-based competitiveness and ultimately selected via feedback from rare taxa.

Overall, our findings reconcile deterministic trait-based assembly rules with the stochastic and context-dependent dynamics driven by rare taxa. Rather than serving as passive background noise, the rare biosphere actively modulates the selection and establishment of dominant taxa. This perspective integrates biotic context into our understanding of dominance, complementing niche-based frameworks that focus on abiotic filtering (32–34). It also builds upon previous insights into microbial interactions—including antimicrobial production (35, 36), antagonism (37), cross-feeding (8, 38), and bacterial cell motility (39)—by emphasizing how low-abundance taxa collectively influence ecological outcomes. Future work may apply this framework to spatially heterogeneous, temporally dynamic, or host-associated environments, where the influence of facilitation, historical contingency, and ecological memory may be even more pronounced. In doing so, this framework may also inform predictive models of community dynamics in applied contexts such as soil restoration, microbiome engineering, or biocontrol.

## MATERIALS AND METHODS

### Collection of raw microbial communities

The rhizosphere soil of *Triticum aestivum* wheat seedlings was collected from an experimental field located in the Yellow River Delta wetlands, Shandong, China. All the wheat roots, along with the adhering soil, were collected and transported to the laboratory within four hours. After removing larger particles and loosely bound soil, the roots, approximately 5 g each, were placed in a 50 mL centrifuge tube filled with 30 mL of phosphate-buffered saline (PBS). The samples were vigorously shaken in a shaker at 180 revolutions per minute for 15 minutes. The roots were subsequently removed, the soil suspension was centrifuged at 5,000 revolutions per minute for 15 minutes to remove the supernatant, and approximately 2 mL of the residual soil suspension was employed as the raw microbial community.

### Dilution and cultivation

A 200 µL volume of the original microbial suspension was mixed with 1.8 mL of 10% TSB, which was vigorously agitated and subsequently subjected to filtration using filter paper, and the resulting refined bacterial suspension was designated as the 1.0 × 10^−1^ dilution. A 200 µL volume of this microbial suspension was mixed with 19.8 mL of PBS, designated the 1.0 × 10^−2^ dilution, from which 10 mL were combined with 90 mL of 10% TSB to generate the 1.0 × 10^−3^ dilution, then to 1.0 × 10^−4^, 0.5 × 10^−4^, and 1.0 × 10^−5^.

Following pretests and guided by prior observations that 30% of turbid wells maximizes the probability of single-cell inoculation per well (40), we selected slightly lower dilution levels (1.0 × 10^−4^, 0.5 × 10^−4^, and 1.0 × 10^−5^) to favor the establishment of simple, low-diversity communities in each well. After pretests, bacterial suspensions were inoculated into 96-well cell culture plates (160 µL per well of the corresponding bacterial suspension), three plates for 1.0 × 10^−4^, four plates for 0.5 × 10^−4^, and four plates for 1.0 × 10^−5^, resulting in a total of 1,056 cultivation wells. These plates were incubated in an incubator at 25°C for 14 days.

### DNA extraction, 16S rRNA gene amplicon sequencing, and analysis pipeline

DNA extraction and sequencing followed a well-established protocol (40). Specifically, DNA was isolated from sub-communities using the alkaline lysis method. The V5–V7 region of the bacterial 16S rRNA gene sequence was amplified using universal primers 799F (5′-AACMGGATTAGATACCCKG-3′) and 1193R (5′-ACGTCATCCCCACCTTCC-3′) (41) in an initial PCR. The first PCR products were diluted and served as the templates for a second PCR, during which each PCR product was labeled with plate and well barcodes, in addition to the Illumina sequencing adapters. The PCR products were then purified using Agencourt AMPure XP beads and sequenced using Illumina technology.

The “Culturome” pipeline (https://github.com/YongxinLiu/Culturome) was assembled using a combination of QIIME (42), USEARCH (43), VSEARCH (44), and other scripts (45, 46) to assess sequence quality, split barcoded sequences, remove the sequencing background, and identify amplicon sequence variants (OTUs). The taxonomic identities of the OTUs and the alpha and beta diversity of the microbial communities were determined using QIIME 2 (47).

### Inference of rRNA gene operon copy number, metabolic gene number, and completeness of metabolic pathway

We separately calculated the rRNA gene copy numbers for the shared, appeared, and disappeared taxa in sub-communities at the family level. For each identified family, we consulted rrnDB (48)—a database containing statistics on rRNA operon copy numbers—to find the median copy number associated with the family.

To explore the metabolic differences of OTUs, we estimated the number of metabolic genes in their genomes using PICRUSt2 (49) with the default settings. We input the 16S rRNA gene sequences of the OTUs along with their abundances in each sub-community. After processing with PICRUSt2, we obtained a table of the predicted gene content for each OTU, specifically noting the presence or absence of specific KEGG orthology (KO) numbers from the kyoto encyclopedia of genes and genomes (KEGG) database. We then filtered this table to extract all metabolic genes, selecting the KO numbers associated with at least one known metabolic reaction. This provided an estimated set of metabolic genes for each OTU, serving as an indirect measure of the OTU metabolic capabilities. Based on these KOs, the completeness of the metabolic KEGG pathway of each OTU was estimated using KEGGDecoder (23).

### Estimate of the possibility of coexistence and metabolic resource overlap of OTU pairs in sub-communities

The POC is an index used to assess the potential for any given OTU to coexist with other OTUs within the same sub-community. It was calculated as follows:


$${\text{The possibility of coexistence (POC) of OTU}}_{x\text{-}y}=\frac{{XY}_{coe}}{X-{XY}_{coe}},$$

where *X* represents the number of sub-community occurrences of OTUx. *XY*coe represents the number of co-occurrences of OTUx and OTUy. OTUy denotes any other possible OTUs, excluding OTUx. There are five potential interaction types between two OTUs: complete coexistence (POC = +∞), coexistence preference (POC > 1), neutral interaction (POC = 1), exclusion preference (0 < POC < 1), and complete exclusion (POC = 0; refer to Fig. S9).

MRO quantifies the similarity of the nutritional requirements between two OTUs (24), which can be employed to reflect the intracommunity risk for resource competition. To calculate the MRO, the 16S rRNA gene sequences of OTUs were mapped to genomes in the NCBI RefSeq database (50) with a 97% identity threshold using BLASTn (51). If multiple genomes were obtained, those with the highest alignment identity were selected. These mapped genomes were used to construct genome-scale metabolic models with CarveMe (52). Based on these metabolic models, the MRO of the selected OTU pairs was calculated using the species metabolic interaction analysis (SMETANA) tool (24).

### Consumer–resource model

To test whether dominant formation is jointly shaped by intrinsic traits and rare taxa interactions, we developed a two-step consumer–resource model (30, 31) that separates ecological nomination from community voting.

Step 1: Trait-based nomination across resource environments

We simulated 400 communities (four resource richness levels × 100 replicates) to evaluate how species with different ecological traits perform under variable resource conditions. Each community consisted of 80 species grouped into four functional types based on growth rate (high vs low) and metabolic flexibility (broad vs narrow): high growth + high metabolic flexibility, high growth + low metabolic flexibility, low growth + high metabolic flexibility, and low growth + low metabolic flexibility (20 species per group). Growth rates and resource utilization breadths were sampled from predefined trait distributions.

Species dynamics were modeled via ordinary differential equations (ODEs) describing biomass accumulation and resource depletion. For each species ii and resource jj, dynamics followed:


$$\frac{dN_{i}}{dt}=N_{i}\left(g_{i}\sum_{j}U_{ij}R_{j}-m\right)$$

$$\frac{dR_{j}}{dt}=-R_{j}\sum_{i}g_{i}N_{i}U_{ij}$$

where *N_i_* and *R_j_* are the abundance of species *i* and concentration of resource *j*, *gi* is the growth rate, U*ij* is the uptake coefficient, and *m* = 0.1 is a uniform maintenance cost. Each species could consume a subset of available resources, sampled to match its metabolic breadth. The uptake matrix *U* was randomly populated for usable resources.

All simulations were initialized with equal biomass across species and run over 100 time units using the odeint solver in SciPy. Final species abundances were grouped to compute relative contributions of each functional type across environments.

Step 2: Context-dependent voting among fixed dominant candidates

To assess how background community composition modulates dominance outcomes, we fixed 20 HH species as dominant candidates and introduced 60 randomly generated rare background species in each of 100 replicates. All HH candidates shared comparable traits and resource profiles across replicates, ensuring uniform intrinsic competitiveness.

Initial biomass was heavily skewed (95% allocated to HH species). The same ODE framework was applied to simulate community assembly. In each replicate, we identified the most abundant species (“Top1”) and recorded which HH candidate succeeded. This allowed us to quantify the variance in dominance outcomes attributable to random differences in rare taxa composition.

All simulations were implemented in Python using NumPy, SciPy, and matplotlib. Species and resource dynamics were solved numerically, and final abundances were rescaled to relative values. Community-level results were visualized via stacked area plots, scatter distributions of relative abundances, and bar plots summarizing how often each HH species became Top1 across replicates.

Step 3: Structured rare taxa with biased resource overlap

To test whether dominance outcomes can be predictably influenced by rare community structure, we designed a structured rare background where all rare taxa exhibited a biased pattern of resource usage. Specifically, in each replicate, 60 rare species were generated such that their metabolic breadths overlapped preferentially with the resource profiles of a subset of HH candidates (10 species). This introduced a consistent ecological interference pressure on part of the HH pool. By contrast, the remaining HH candidates (the other 10 species) received less direct competition from the rare background.

This setup simulates a “voting” scenario, where rare taxa collectively modulate competitive dynamics via asymmetric resource overlap. Importantly, the HH candidate pool remained identical across replicates, ensuring that any variation in dominance outcomes could be attributed solely to background-mediated interactions rather than intrinsic differences among candidates.

Simulations followed the same consumer–resource model as in Step 2. For each of 100 replicates, we identified the Top1 species and compared its identity between structured and random background scenarios. Bar plots were used to visualize shifts in the frequency distribution of HH species becoming Top1, highlighting the extent to which structured interference influenced competitive outcomes. This context-dependent shift provides mechanistic support for a nomination–voting framework in microbial dominance assembly.

## Data Availability

All raw sequencing data have been deposited in the NCBI Sequence Read Archive (SRA) under accession numbers SRR27666106 and SRR27666108. Custom codes used in this study are available on GitHub (https://github.com/wangjianing0618/microbial-community-before-and-after-dilution-cultivation).

## SUPPLEMENTAL MATERIAL

Supplemental figures mbio.02598-25-s0001.docx Fig. S1 to S13.

Supplemental tables mbio.02598-25-s0002.xlsx Table S1 to S13.

ASM does not own the copyrights to Supplemental Material that may be linked to, or accessed through, an article. The authors have granted ASM a non-exclusive, world-wide license to publish the Supplemental Material files. Please contact the corresponding author directly for reuse.
