# Supplementary material for: Rare taxa modulate the emergence of dominants in microbial communities
Source: mBio. 2025 Nov 25;17(1):e02598-25. doi: 10.1128/mbio.02598-25 (PMC12802277; doi:10.1128/mbio.02598-25)
Supplement: Supplemental figures — Fig. S1 to S13. [file mbio.02598-25-s0001.docx]

**Supplementary information for**

**Rare taxa modulate the emergence of dominants in microbial communities**

Jianing Wang^1^*, Jianshui Yu^1^, Zhuo Pan^1^, Zheng Zhang^1^, Yue-zhong Li^1^*

1. *State Key Laboratory of Microbial Technology, Institute of Microbial Technology, Shandong University, Qingdao 266237, P. R. China*

*Corresponding authors. Emails: wangjianing@sdu.edu.cn; lilab@sdu.edu.cn.

**Supplementary Figures**

**Fig. S1. Rarefaction curves illustrate the dependence of observed OTUs on sequencing depth. a**, Raw microbial communities. **b**, Cultivated sub-communities.

**Fig. S2. OTUs detected in the raw** **microbial communities from *Triticum aestivum* rhizosphere. a**, The rank–abundance curves of all OTUs. **b**, The dominant and rare OTU numbers. **c**, The relative abundance of dominant and rare OTUs. The dominant and rare OTUs are designated by the 1.0% relative abundance threshold.

**Fig. S3. Raw reads of all sub-communities.** The sub-communities containing > 5000 reads were selected for following analysis. The selected 908 sub-communities included 195 of 1.0 × 10^-4^, 355 of 0.5 × 10^-4^ and 358 of 1.0 × 10^-5^ dilution.

**Fig. S4. Number of observed OTUs in sub-communities.**

**Fig. S5. Taxonomic compositions of shared, appeared and disappeared OTUs in raw- and sub-communities.** **a**, Class. **b**, Order. **c**, Genus.

**Fig. S6. Relationships between the appearing number and the frequency to become dominant or rare in sub-communities. a**, The threshold for dominant taxa: 0.5%. **b**, The threshold for dominant taxa: 0.1%.

**Fig. S7. Changes of the number of D-to-D, R-to-D, D-to-R and R-to-R OTUs along with the observed OTUs.** D-to-D: the OTUs from dominant to dominant; R-to-D: rare to dominant, D-to-R: dominant to rare, R-to-R: rare to rare.

**Fig. S8. Correlation analysis of observed OTUs, D-to-D OTUs, D-to-R OTUs, R-to-D OTUs and R-to-R OTUs.**

**Fig. S9. The possibility of coexistence (POC) of OTU pair.**

**Fig. S10. The appearing number of dominant OTUs in sub-communities.**

**Fig. S11. Relative abundance distribution of Top1OTU in sub-communities.**

**Fig. S12. PCoA based on Bray-Curtis metric results (OTU level) of sub-communities with the highlight of those containing OTU_1 (a), OTU_2 (b), OTU_3 (c) and the Top1OTUs (d) according to their relative abundance.**

**Fig. S13. Stacked scatter plot showing the relative abundance of each High growth + High flexibility (HH) species across 100 trials. HH species serve as fixed dominant candidates in the voting phase.**

**Supplementary Tables**

**Table S1. OTUs detected in raw-communities of *Triticum aestivum*.**

**Table S2. Sequencing details of the 908 sub-communities from *Triticum aestivum*.**

**Table S3. Alpha diversity indices of sub-communities from *Triticum aestivum*.**

**Table S4. Shared, disappeared and appeared OTUs in sub-communities of *Triticum aestivum*.**

**Table S5. Taxonomic composition of shared, appeared and disappeared OTUs.**

**Table S6. Fate and growth metabolic potential of each OTU.**

**Table S7. The relative abundance and number of OTUs in sub-communities.**

**Table S8. The possibility of coexistence (POC) of OTU pairs in sub-communities.**

**Table S9. The metabolic resource overlap (MRO) analysis of the top 100 OTU pairs with the highest occurrence frequency from each of the DD_coe_, DR_coe_, RD_coe_ and RR_coe_ pairs.**

**Table S10. Information and relative abundance of the top five most abundant OTUs in sub-communities from *Triticum aestivum*.**

**Table S11. Taxonomic compositions and distribution (sub-communities of D-to-D > D-to-R or D-to-D < D-to-R) of Top1OTUs in sub-communities of *Triticum aestivum*.**

**Table S12. Relative abundance of dominant OTUs (D-to-D or R-to-D) and Top1OTU information of each sub-community.**

**Table S13. Results of pairwise PERMANOVA among sub-communities stratified by Top1OTUs.**


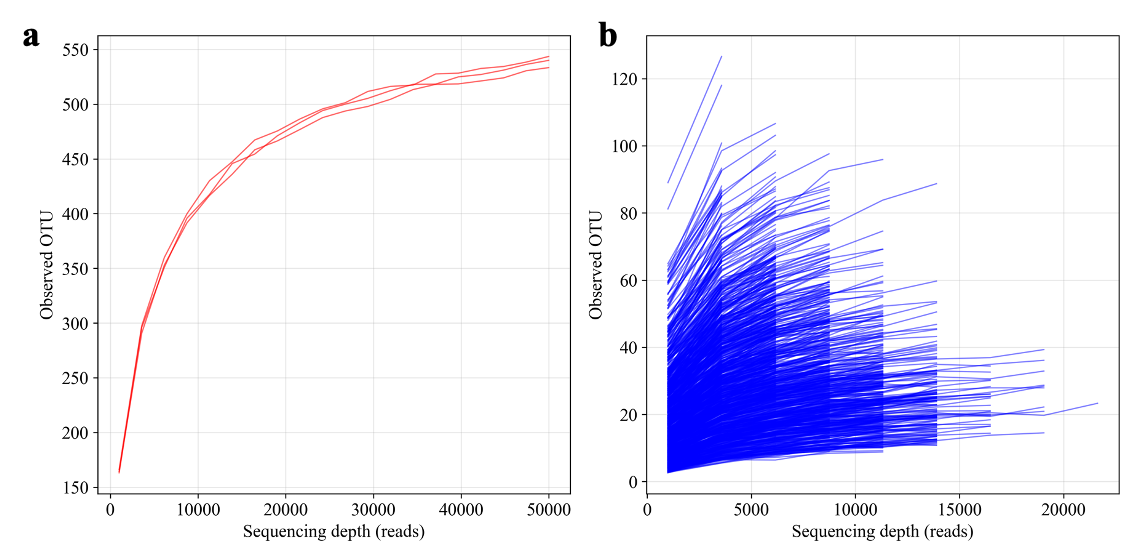


**Fig. S1. Rarefaction curves illustrate the dependence of observed OTUs on sequencing depth. a**, Raw microbial communities. **b**, Cultivated sub-communities.

**
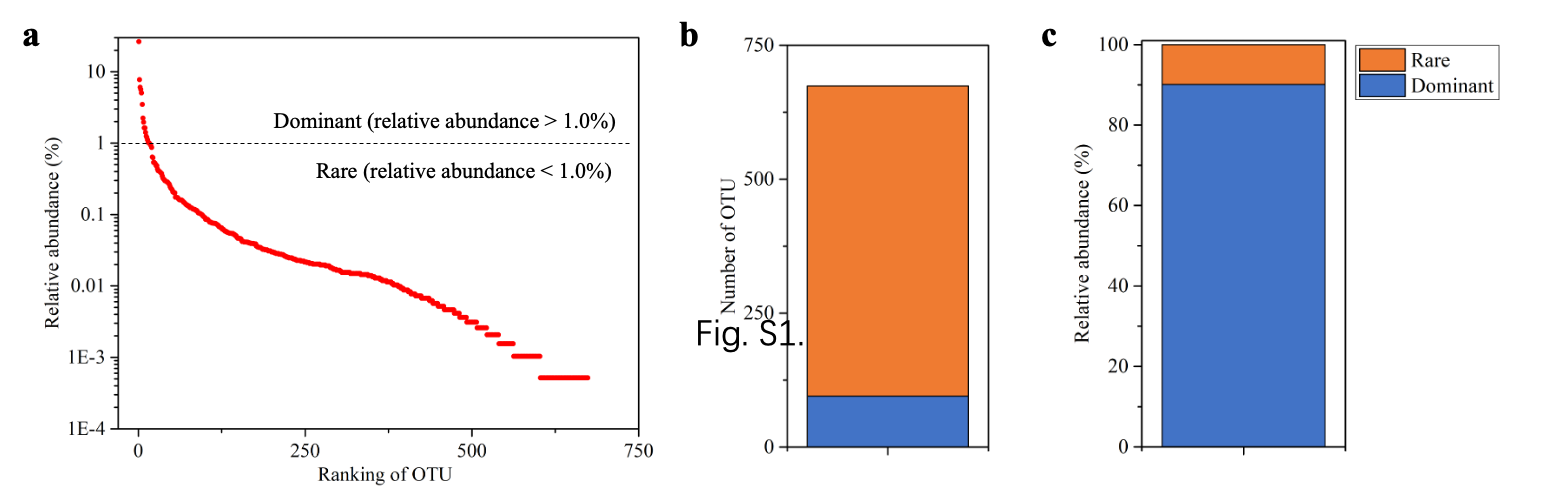
**

**Fig. S2. OTUs detected in the raw microbial communities from *Triticum aestivum* rhizosphere. a**, The rank–abundance curves of all OTUs. **b**, The dominant and rare OTU numbers. **c**, The relative abundance of dominant and rare OTUs. The dominant and rare OTUs are designated by the 1.0% relative abundance threshold.


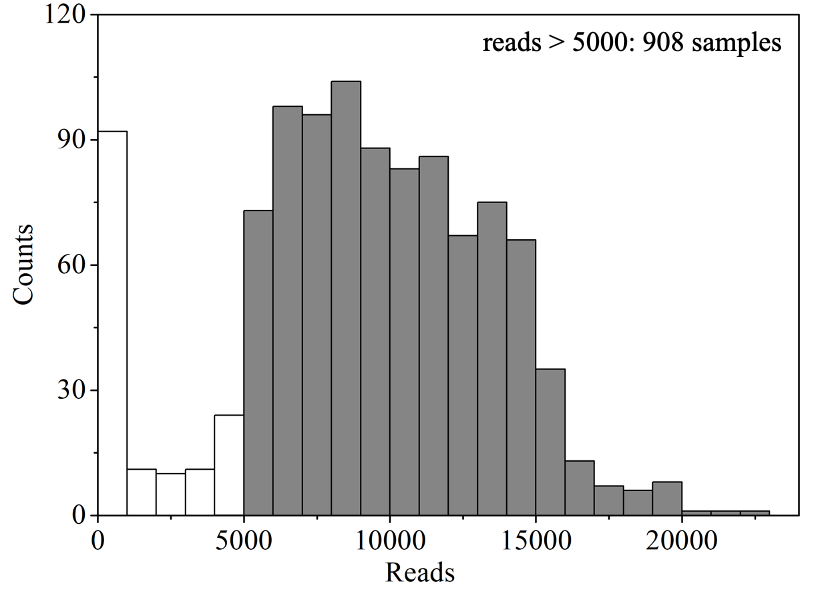


**Fig. S3. Raw reads of all sub-communities.** The sub-communities containing > 5000 reads were selected for following analysis. The selected 908 sub-communities included 195 of 1.0 × 10^-4^, 355 of 0.5 × 10^-4^ and 358 of 1.0 × 10^-5^ dilution.


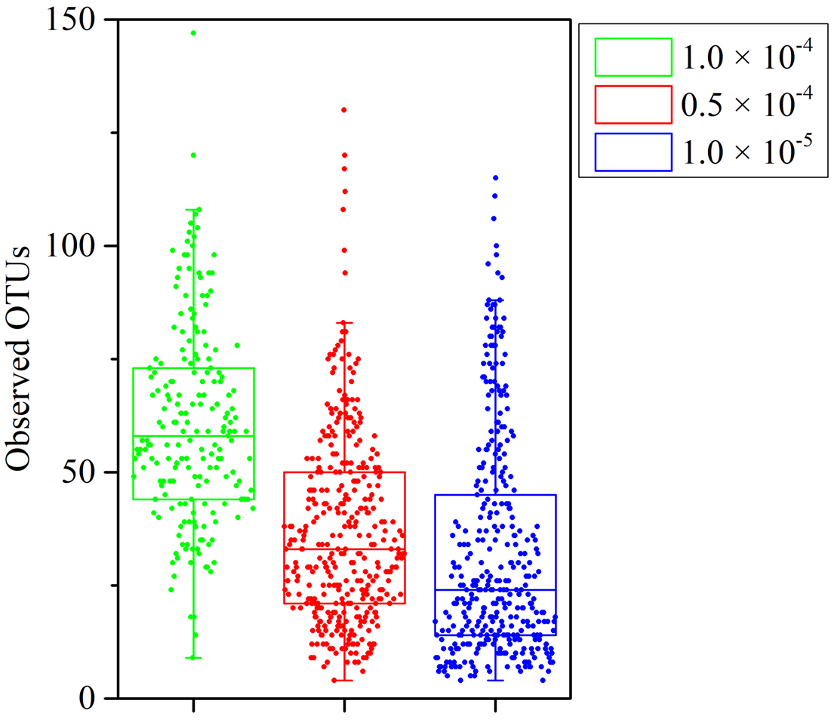


**Fig. S4. Number of observed OTUs in sub-communities.**

**
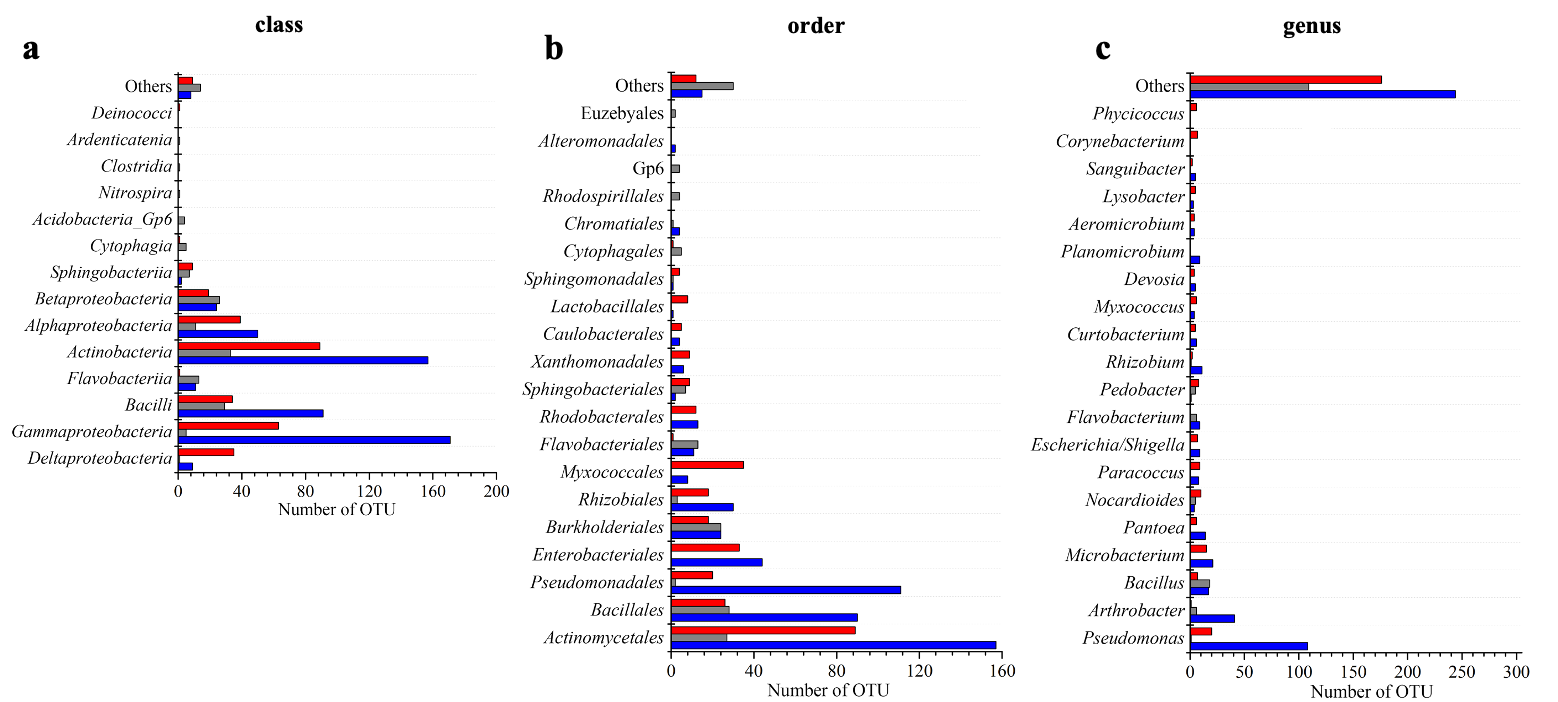
**

**Fig. S5. Taxonomic compositions of shared, appeared and disappeared OTUs in raw- and sub-communities. a**, Class. **b**, Order. **c**, Genus.

**
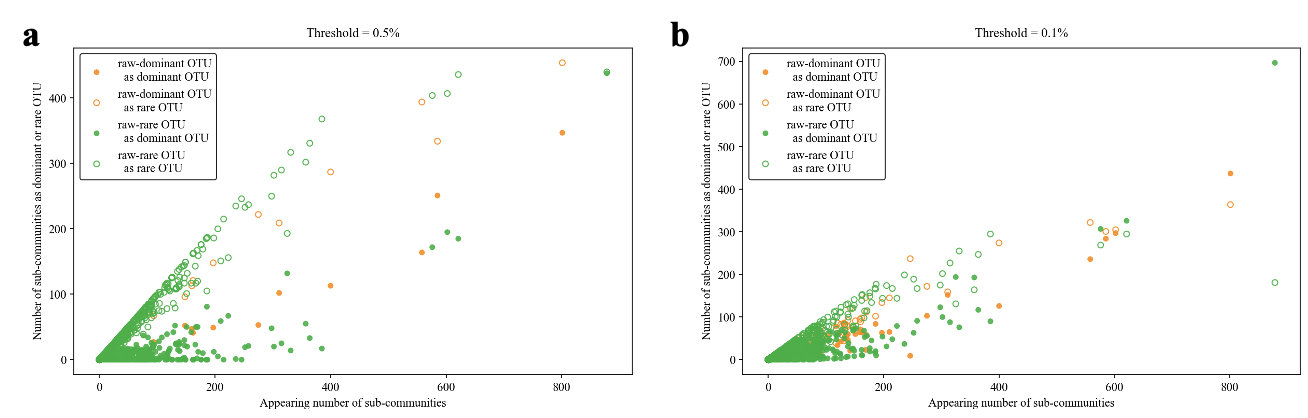
**

**Fig. S6. Relationships between the appearing number and the frequency to become dominant or rare in sub-communities. a**, The threshold for dominant taxa: 0.5%. **b**, The threshold for dominant taxa: 0.1%.


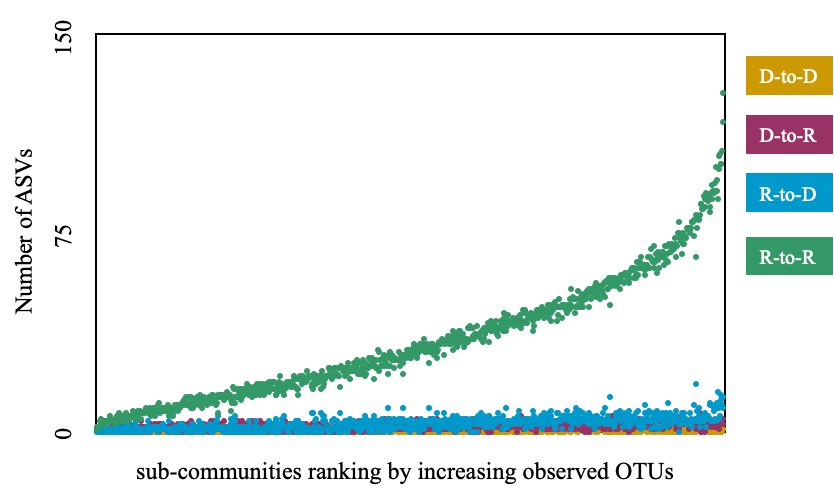


**Fig. S7. Changes of the number of D-to-D, R-to-D, D-to-R and R-to-R OTUs along with the observed OTUs.** D-to-D: the OTUs from dominant to dominant; R-to-D: rare to dominant, D-to-R: dominant to rare, R-to-R: rare to rare.

**
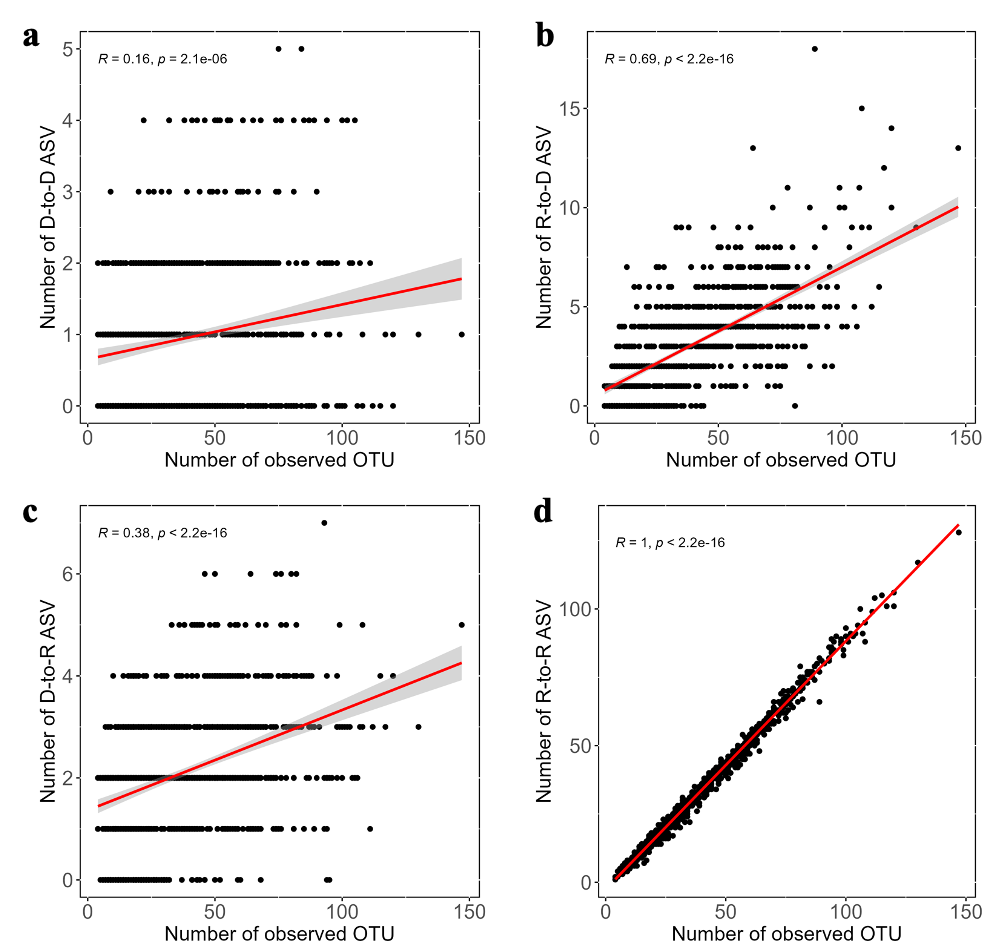
**

**Fig. S8. Correlation analysis of observed OTUs, D-to-D OTUs, D-to-R OTUs, R-to-D OTUs and R-to-R OTUs.**


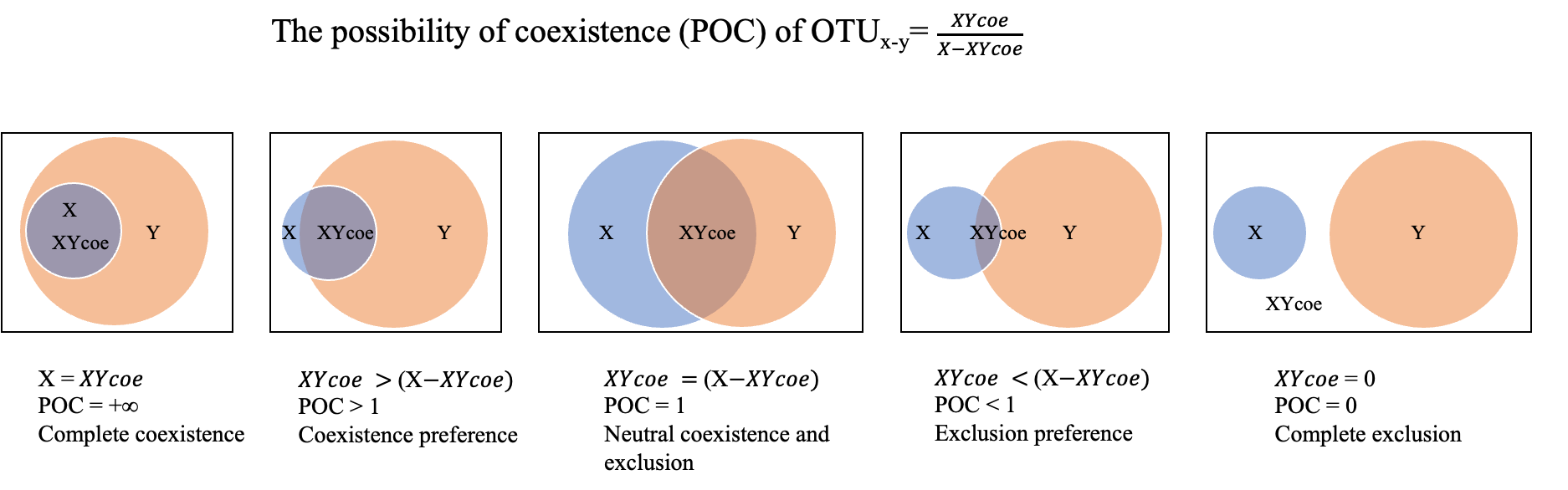


**Fig. S9. The possibility of coexistence (POC) of OTU pair.**


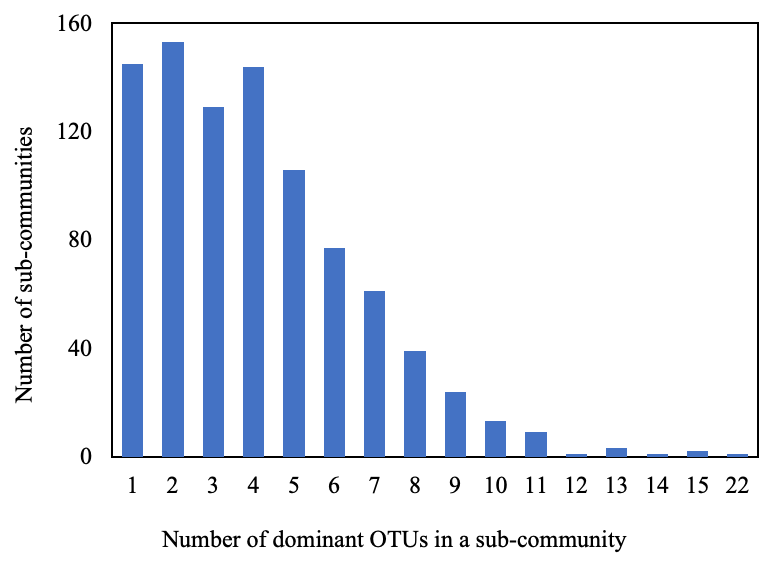


**Fig. S10. The appearing number of dominant OTUs in sub-communities.**


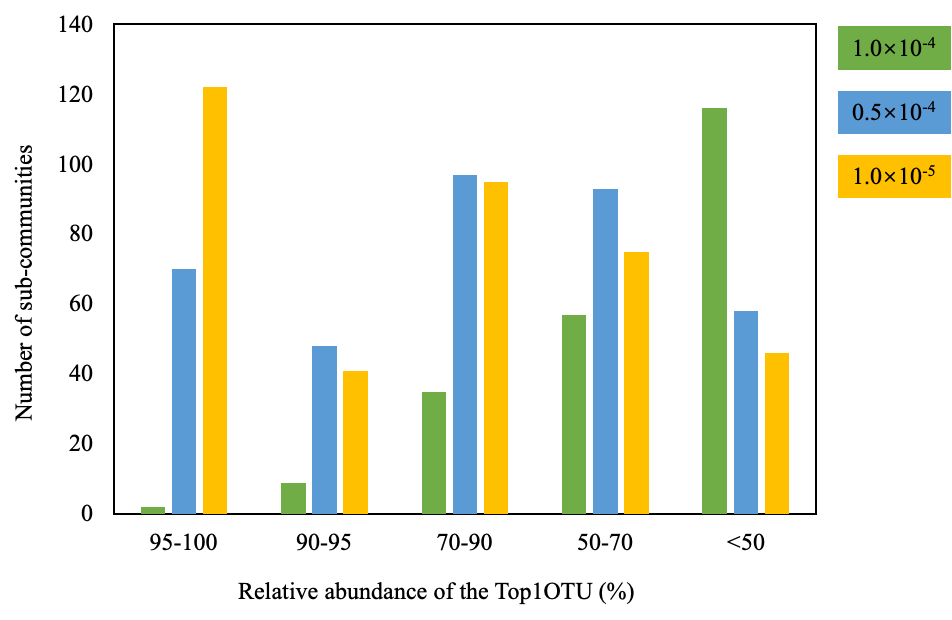


**Fig. S11. Relative abundance distribution of Top1OTU in sub-communities.**

**
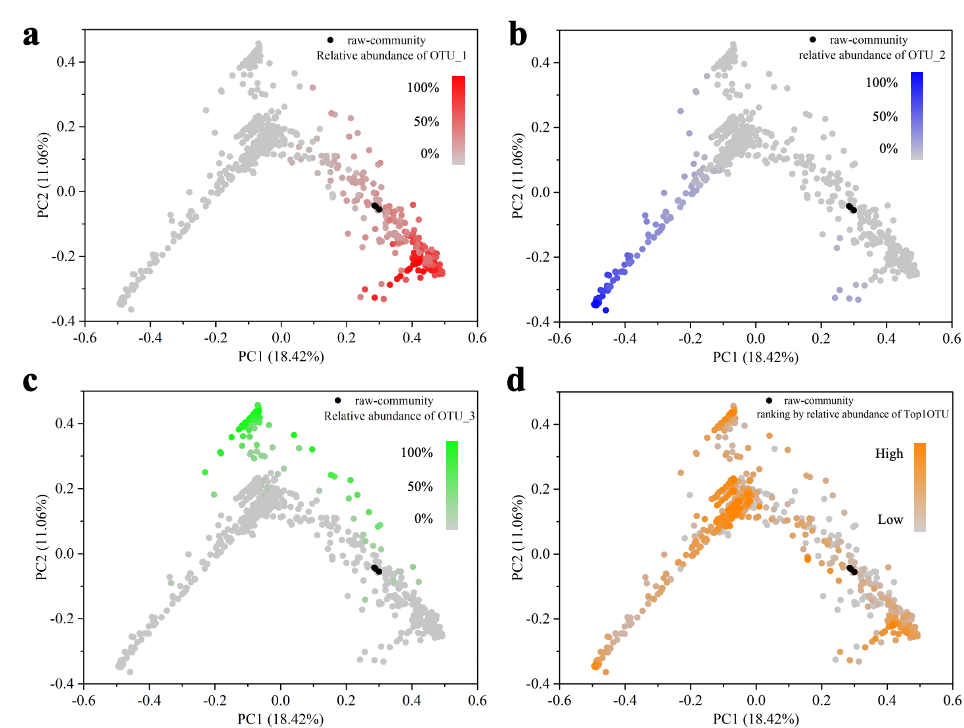
**

**Fig. S12. PCoA based on Bray-Curtis metric results (OTU level) of sub-communities with the highlight of those containing OTU_1 (a), OTU_2 (b), OTU_3 (c) and the Top1OTUs (d) according to their relative abundance.**


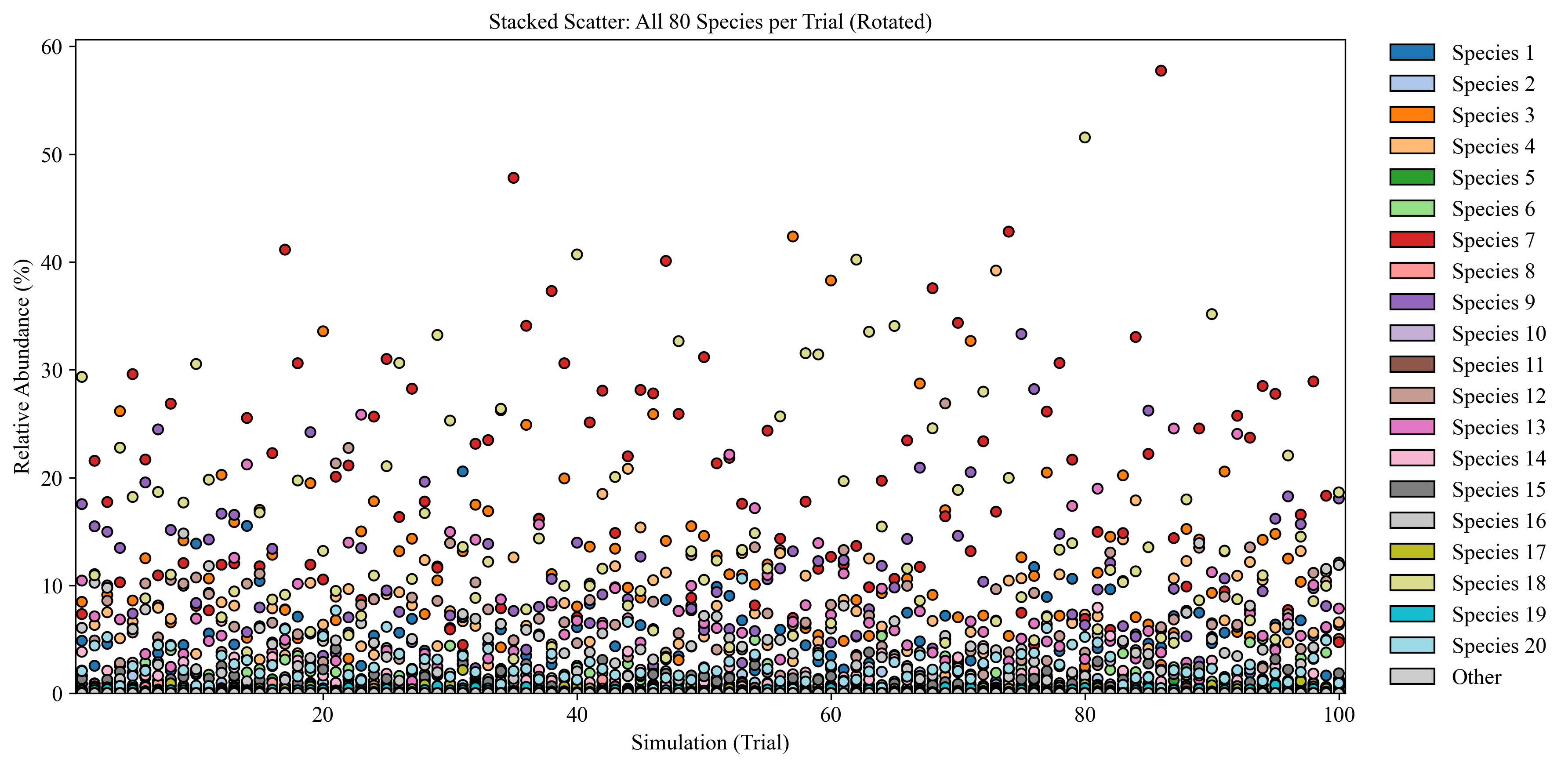


**Fig. S13. Stacked scatter plot showing the relative abundance of each High growth + High flexibility (HH) species across 100 trials. HH species serve as fixed dominant candidates in the voting phase.**
